# Value of the high-sensitivity troponin T assay for diagnosis of acute myocardial infarction in patients with and without renal insufficiency

**DOI:** 10.1080/0886022X.2020.1845732

**Published:** 2020-11-12

**Authors:** Cheng-Kai Hsu, I-Wen Wu, Yih-Ting Chen, Chia-Huei Peng, Yi-Ju Tseng, Yung-Chang Chen, Ming-Jui Hung, Yu-Cheng Kao

**Affiliations:** a Department of Nephrology, Chang Gung Memorial Hospital, Keelung, Taiwan; b College of Medicine, Chang Gung University, Taoyuan, Taiwan; c Department of Pediatric Gastroenterology and Nutrition, Mackay Children’s Hospital, Taipei, Taiwan; d Department of Information Management, Chang Gung University, Taoyuan, Taiwan; e Department of Laboratory Medicine, Chang Gung Memorial Hospital, Taoyuan, Taiwan; f Healthy Aging Research Center, Chang Gung University, Taoyuan, Taiwan; g Departments of Cardiology and Community Medicine Research Center, Chang Gung Memorial Hospital, Keelung

**Keywords:** Acute myocardial infarction (AMI), chronic kidney disease (CKD), renal failure, troponin T

## Abstract

**Background:**

Cardiac troponins are important markers for diagnosis of acute myocardial infarction (AMI) in general population; however, chronically-elevated troponins levels are often seen in patients with renal insufficiency, which reduce their diagnostic accuracy. The aim of our study was to access the diagnostic values of initial high-sensitive cardiac troponin T (hs-cTnT) and relative change of hs-cTnT for AMI in patients with and without renal insufficiency.

**Methods:**

Cardiac care unit patients with elevated hs-cTnT levels in 2017–2018 were enrolled. Receiver operating characteristic (ROC) curves were used to evaluate initial hs-cTnT levels and relative changes after 3 h of enrollment for diagnosis of AMI in patients with estimated glomerular filtration rate (eGFR) < 60 mL/min/1.73 m^2^ (low), and eGFR ≥ 60 mL/min/1.73 m^2^ (normal).

**Results:**

Of 359 patients, 240 patients had low eGFR, and 119 patients had normal eGFR. The area under the ROC curve (AUC) for the initial hs-cTnT levels was 0.58 (95% CI, 0.5–0.65, *p* = 0.053) among patients with low eGFR and 0.54 (95% CI, 0.4–0.67, *p* = 0.612) among patients with normal eGFR. AUCs for relative changes of hs-cTnT were 0.82 (95% CI, 0.76–0.88, *p* < 0.001) in patients with low eGFR and 0.82 (95% CI, 0.71–0.91, *p* < 0.001) in patients with normal eGFR. Optimal cutoff values for the relative changes in hs-cTnT were 16% and 12% in patients with low eGFR and normal eGFR, respectively.

**Conclusions:**

Relative changes in hs-cTnT levels had better diagnostic accuracy than initial hs-cTnT levels.

## Introduction

Acute myocardial infarction (AMI) is a major cause of mortality and disability around the world [1]. Chronic kidney disease (CKD) is an independent risk factor for AMI and the risk increases with advanced renal insufficiency [2,3]. Additionally, AMI patients comorbid with CKD are not ideal patients for coronary angiography and percutaneous coronary intervention, therefore having higher mortality rates than AMI patients without CKD [2,4–8]. Furthermore, AMI patients comorbid with advanced CKD have a higher rate of long-term adverse cardiac events than those with normal kidney function [9]. Coronary revascularization in the case of an AMI is associated with lower in-hospital mortality and 12-month mortality versus conservative management in patients with CKD [2,4,8,10]. However, the key concern in performing coronary angiography in CKD patients is the risk of contrast-induced toxicity, subsequent renal deterioration, and adverse long-term outcomes [11]. On the other hand, an indolent clinical course, atypical presentation, and unspecific or false-positive elevation of cardiac enzymes which often present in CKD patients all contribute to the delay in the diagnosis or the reperfusion of damaged myocardium. Therefore, timely and accurate diagnoses of AMI in patients with CKD are crucial to reduce unnecessary exposure to contrast media in coronary angiography. Cardiac troponin T and troponin I are important markers for the diagnosis of AMI in the general population [1]. The development of high-sensitivity cardiac troponin testing allows for the detection of small amounts of myocardial necrosis at the expense of a loss of specificity. For patients with chronic elevation of cardiac troponin without MI, troponin T or troponin I tend not to change acutely over time. The authors of a previous review have recommended that a relative change in cardiac troponin levels of more than 20% should be used to diagnose AMI in patients undergoing dialysis who have symptoms such as chest pain or dyspnea [12]. However, no consensus has been established for the optimal cutoff value of a relative change in high-sensitivity cardiac troponin T (hs-cTnT) for the diagnosis of AMI in patients with renal insufficiency. We investigated the diagnostic accuracy of the initial measurements of hs-cTnT at baseline and the relative changes in this enzyme 3-h after initial data for the diagnosis of AMI in patients with and without current renal insufficiency and in patients with and without preexisting CKD.

## Materials and methods

In this prospective study, all patients 18 years or older admitted to the cardiac care unit (CCU) of Chang Gung Memorial Hospital at Keelung with elevated hs-cTnT T (> 99^th^ percentile of the upper reference range) and clinical manifestations suspicious of angina (such as typical chest pain, dyspnea, upper abdominal pain, change in consciousness, and cardiac arrest) were enrolled from 1 September 2017 through 30 June 2018. Change in consciousness was defined as decreased score of Glasgow Coma Scale ≥1 and without other identifiable symptoms [13]. Patients who had diagnoses of atrioventricular block, pulmonary embolism, and infective endocarditis were excluded. The Institutional Review Board at Chang Gung Memorial Hospital (201900289B0) approved this study.

Information was collected for further analyses, including demographic variables and previous comorbid conditions (diabetes, hypertension, coronary artery disease, heart failure, cerebrovascular accident, peripheral arterial occlusive disease, liver cirrhosis, malignancy, and chronic obstructive pulmonary disease). In addition, clinical biochemistry was assessed upon the first day of hospitalization. These parameters included serum urea nitrogen, creatinine, estimated glomerular filtration rate (eGFR), sodium, potassium, chloride, calcium, phosphate, magnesium, hemoglobin, albumin, lipid profile, glycohemoglobin, and hs-cTnT.

We measured hs-cTnT on enrollment and repeated 3-h later. All assays were performed on the Cobas-E601 analyzer using an electrochemiluminescence (ECLIA) assay (Roche Diagnostics, Mannheim, Germany) with a limit of detection of 3 ng/L. The 99^th^ percentile cutoff point was 14 ng/L, and the coefficient of variation of 10% was 13 ng/L. The eGFR was calculated by use of the Chronic Kidney Disease Epidemiology Collaboration formula. CKD was defined as an eGFR <60 mL/min/1.73 m^2^ or any proteinuria on two separate occasions more than three months apart [14].

All the patients were suggested to receive coronary angiography within 3 days of admission for diagnostic and therapeutic purposes. Coronary angiography were only performed in those with informed consent from the patients or their families. For patients who underwent coronary angiography, AMI was defined as having at least one of the following findings in the coronary arteries: (1) > 70% stenosis, (2) thrombus, (3) unstable plaque with or without rupture, and (4) intimal damage. For patients who did not undergo coronary angiography, AMI was defined as echocardiographic regional wall motion abnormalities and new, significant ST–segment-T wave changes on electrocardiography.

Descriptive statistics were expressed as mean ± standard deviation (SD) or median (interquartile range, IQR). Discrete variables were presented as frequency and percentage. The Kolmogorov-Smirnov method was used to test for the normality of numerical variables. Differences in continuous variables between the two groups were analyzed using the Student t-test or Mann Whitney U test. Differences in categorical variables between the two groups were compared using the chi-square test. We constructed receiver operating characteristic (ROC) curves and evaluated the area under the curve (AUC) to estimate the predictive accuracy of baseline hs-cTnT and the relative changes of hs-cTnT in 3-h. We compared the above results between patients with eGFR < 60 mL/min/1.73 m^2^ (low) and those with eGFR ≥ 60 mL/min/1.73 m^2^ (normal), and also compared the above results between patients with and without a history of CKD [CKD (+), CKD (−)]. The statistical differences between 2 ROC curves were calculated by DeLong test [15]. Cutoff values based on the ROC curves were used to calculate the sensitivity and specificity of the hs-cTnT test for the diagnosis of AMI in the two patient groups. The optimal cutoff values had the highest combined sensitivity and specificity in each group.

The statistical analyses were performed using SPSS, version 21.0 (IBM, Armonk, NY). All statistical tests were two-tailed, and a *p* value < 0.05 was considered statistically significant.

## Results

**Figure 1.**
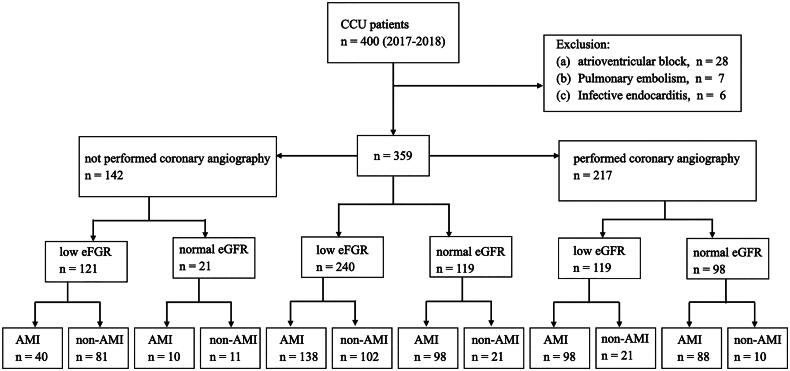
Enrollment flowchart and patient status. Low eGFR was defined as eGFR < 60 mL/min/1.73m^2^ and normal eGFR was defined as eGFR ≥ 60 mL/min/1.73m^2^. Abbreviations: CCU, cardiac care unit; eGFR, estimated glomerular filtration rate; AMI, acute myocardial infarction.

There were 400 patients with elevated hs-cTnT levels admitting to CCU. We excluded patients with atrioventricular block (*n* = 28), pulmonary embolism (*n* = 7), and infective endocarditis (*n* = 6). Finally, 359 patients were enrolled in this prospective study (Figure 1). The mean patient age was 69.5 ± 13.8 years; 223 (62.1%) were men, and 136 were women (37.9%). The median time between sampling of hs-cTnT and onset of symptoms was 3 h (IQR: 2–5 h). Table 1 summarizes the baseline characteristics of the patients. Patients with low eGFR were older (72.7 ± 12.8 versus 63.1 ± 13.6 years old, *p* < 0.001), were less likely to be men (57.1 versus 72.3%, *p* = 0.006) and fewer smoked tobacco (32.9 versus 47.9%, *p* = 0.01) than those patients with normal eGFR. Patients with low eGFR had higher prevalence of diabetes (52.1 versus 35.3%, *p* = 0.003), hypertension (74.2 versus 52.9%, *p* < 0.001), coronary artery disease (34.6 versus 21.8%, *p* = 0.014), heart failure (34.2 versus 12.6%, *p* < 0.001), cerebrovascular accident (18.8 versus 8.5%, *p* = 0.011), and peripheral arterial occlusive disease (4.2 versus 0%, *p* = 0.025) than patients with normal eGFR.

**Table 1.** Demographic and clinical characteristics of patients with moderate to severe renal insufficiency and normal kidney function.

Characteristic	All patients (*n* = 359) No. (%)^b^	eGF*R* < 60 mL/min/1.73 m^2^ (*n* = 240) No. (%)^b^	eGF*R* ≥ 60 mL/min/1.73 m^2^ (*n* = 119) No. (%)^b^	*p* Value
Age, mean ± SD (yr)	69.5 ± 13.8	72.7 ± 12.8	63.1 ± 13.6	<0.001
Men	223 (62.1)	137 (57.1)	86 (72.3)	0.006
BMI, mean ± SD (kg/m^2^)	24 ± 4.4	23.7 ± 4.5	24.6 ± 4.1	0.097
Smoking	136 (37.8)	79 (32.9)	57 (47.9)	0.01
Primary disease				
Diabetes	167 (46.5)	125 (52.1)	42 (35.3)	0.003
Hypertension	241 (67.1)	178 (74.2)	63 (52.9)	<0.001
CAD	109 (30.4)	83 (34.6)	26 (21.8)	0.014
HF	97 (27)	82 (34.2)	15 (12.6)	<0.001
CVA	55 (15.4)	45 (18.8)	10 (8.5)	0.011
PAOD	10 (2.8)	10 (4.2)	0 (0)	0.025
Liver cirrhosis	9 (2.5)	7 (2.9)	2 (1.7)	0.723
Malignancy	31 (8.7)	21 (8.8)	10 (8.5)	0.931
COPD	32 (8.9)	25 (10.4)	7 (5.9)	0.156
Initial findings^c^				
BUN, median (IQR) (mEq/L)	31.3 (19.5–52.1)	37.6 (26.8–57.1)	16.5 (13.5–21.6)	<0.001^a^
Cr, median (IQR) (mEq/L)	1.4 (1.0–2.3)	1.9 (1.4–3.6)	0.89 (0.8–1.0)	<0.001^a^
eGFR, median (IQR) (mL/min/1.73 m^2^)	53 (30.6–72.5)	35.2 (22.9–48.1)	76 (67.9–94)	<0.001^a^
Na (mEq/L)	136.9 ± 9.1	136.4 ± 10.2	138 ± 6.1	0.103
K (mEq/L)	4.3 ± 1	4.5 ± 1	4 ± 0.6	<0.001
Cl (mEq/L)	99.3 ± 7.1	99.1 ± 7.4	100 ± 5.5	0.669
Ca (mg/dL)	8 ± 1	8.9 ± 1.1	8.8 ± 0.7	0.889
P (mg/dL)	4.8 ± 2.3	4.9 ± 2.4	4 ± 2.1	0.125
Mg, median (IQR) (mEq/L)	1.7 (1.6–1.9)	1.8 (1.6–2)	1.7 (1.6–1.8)	0.001^a^
Hemoglobin (g/dL)	12.2 ± 2.9	11.4 ± 2.9	13.7 ± 2.3	<0.001
Albumin (g/dL)	3.6 ± 0.6	3.6 ± 0.5	3.8 ± 0.6	<0.001
Total cholesterol (mg/dL)	164.3 ± 50.8	151.7 ± 47.1	179.6 ± 51.2	<0.001
TG, median (IQR) (mg/dL)	113.5 (79–160)	98 (77.3–154.8)	125.5 (85.8–169)	0.054^a^
LDL (mg/dL)	105.9 ± 47	93.8 ± 41.6	120.5 ± 49.1	<0.001
HDL (mg/dL)	43.3 ± 15.8	42.2 ± 15.8	44.6 ± 15.7	0.256
HbA1c (%)	6.9 ± 1.66	6.8 ± 1.6	6.9 ± 1.7	0.515
Troponin-T, median (IQR) (ng/L)	142.3 (53.4–385)	160.4 (74–415.3)	97.7 (28–366.2)	0.002^a^
Relative changes in troponin-T at 3-hours, median (IQR) (%)	42.6 (2.7–361.2)	21.7 (1–142.5)	204.9 (38.5–2712)	<0.001^a^

^a^*p* Value using Mann-Whitney U test.

^b^All data are No. (%) unless otherwise indicated.

^c^Data are mean ± SD unless otherwise indicated.

eGFR: estimated glomerular filtration rate; BMI: body mass index; CAD: coronary artery disease; HF: heart failure; CVA: cerebral vascular accident; PAOD: peripheral arterial occlusive disease; COPD: chronic obstructive pulmonary disease; BUN: blood urea nitrogen; Cr: creatinine; TG: triglycerides; LDL: low-density lipoprotein; HDL: high-density lipoprotein; HbA1c: glycohemoglobin.

Initial laboratory studies revealed that patients with low eGFR had higher serum potassium (4.5 ± 1 versus 4 ± 0.6 mEq/L, *p* < 0.001), higher serum magnesium [median (IQR) 1.8 (1.6-2) versus 1.7 (1.6–1.8) mEq/L, *p* = 0.001], lower hemoglobin (11.4 ± 2.9 versus 13.7 ± 2.3 g/dL, *p* < 0.001), lower serum albumin (3.55 ± 0.53 versus 3.84 ± 0.6 g/dL, *p* < 0.001), lower total cholesterol (151.7 ± 47.1 versus 179.6 ± 51.2 mg/dL, *p* < 0.001), lower low-density lipoprotein (93.78 ± 41.62 versus 120.46 ± 49.13 mg/dL, *p* < 0.001) and higher baseline hs-cTnT levels [median (IQR) 160.4 (74–415.3) versus 97.7 (28–366.2) ng/L, *p* = 0.002] than patients with normal eGFR.

Table 2 compares the initial symptoms of AMI between the two groups. For patients with low eGFR, the most common symptom was dyspnea (57.5%), whereas chest pain (73.9%) was the most common symptom for those with normal eGFR. Of the 240 patients with low eGFR who had initial hs-cTnT levels above the normal range (> 14 ng/L), 102 (42.5%) had pathologies other than AMI. Table 3 shows an overview of the potential causes for the elevated hs-cTnT. Among the patients with final diagnosis of AMI, those with low eGFR had higher baseline hs-cTnT levels [median (IQR), 180.2 (76.5–645.3) versus 100.7 (28.2–526.3) ng/L, *p* = 0.002] than those with normal eGFR.

**Table 2.** Clinical presentation of patients having elevated high-sensativity troponin-T.

Presentation	Overall (*n* = 359) No. (%)	eGFR (mL/min/1.73 m^2^)	
		<60 (*n* = 240) No. (%)	≥60 (*n* = 119) No. (%)
Dyspnea	168 (46.8)	138 (57.5)	30 (25.2)
Orthopnea	115 (32)	97 (40.4)	18 (15.1)
Chest pain	192 (53.5)	104 (43.3)	88 (73.9)
Epigastric pain	16 (4.5)	12 (5)	4 (3.4)
Consciousness change	24 (6.7)	18 (7.5)	6 (5)
Cardiac arrest	32 (8.9)	24 (10)	8 (6.7)

**Table 3.** Final diagnoses of patients admitted to CCU and the corresponding levels of high-sensitivity troponin-T.

	Overall (%), *n* = 359	eGFR (mL/min/1.73 m^2^)		*p* Value*
		< 60 (%), *n* = 240	≥ 60 (%), *n* = 119	
AMI	236 (65.7)	138 (57.5)	98 (82.4)	
Initial troponin-T, median (IQR) (ng/L)	144.4 (50.1–614.3)	180.2 (76.5–645.3)	100.7 (28.2–526.3)	0.002
Relative changes in troponin-T at 3-hours, median (IQR) (%)	132.1 (30.1–866)	77.9 (21.8–404.5)	256.5 (64.1–5722.5)	<0.001
Non-AMI	123 (34.3)	102 (42.5)	21 (17.6)	
Heart failure	97 (27)	82 (34.2)	15 (12.6)	
Initial troponin-T, median (IQR) (ng/L)	138.6 (50.2–250.3)	143.2 (56.7–260)	30.1 (17.9–214.5)	0.193
Relative change in troponin-T at 3-hours, median (IQR) (%)	2.7 (−3.5–10.9)	2.6 (−3.3–10.4)	12 (−12–410.4)	0.365
Stroke	5 (1.4)	2 (0.8)	3 (2.5)	
Initial troponin-T, median (IQR) (ng/L)	94.6 (35.2–139.4)	71.7 (30.4 − 113)	94.6 (39.9–165.8)	0.564
Relative change of troponin-T at 3-hours, median (IQR) (%)	−13.3 (−15.3–1)	5939 (1 − 11876)	−15.3 (−17.6–−13.3)	0.121
Sepsis	21 (5.9)	18 (7.5)	3 (2.5)	
Initial troponin-T, median (IQR) (ng/L)	260 (131.4––450)	256.2 (130–450.4)	409.9 (212.6–462.6)	0.421
Relative change in troponin-T at 3-hours, median (IQR) (%)	−0.4 (−9.7–24)	−0.5 (−8.2–3.8)	42.2 (−12–47.2)	0.314

*p Value: eGFR < 60 versus > 60 mL/min/1.73 m^2^ applying the Mann-Whitney U test.

CCU: cardiac care unit; eGFR: estimated glomerular filtration rate; AMI: acute myocardial infarction.

**Figure 2.**
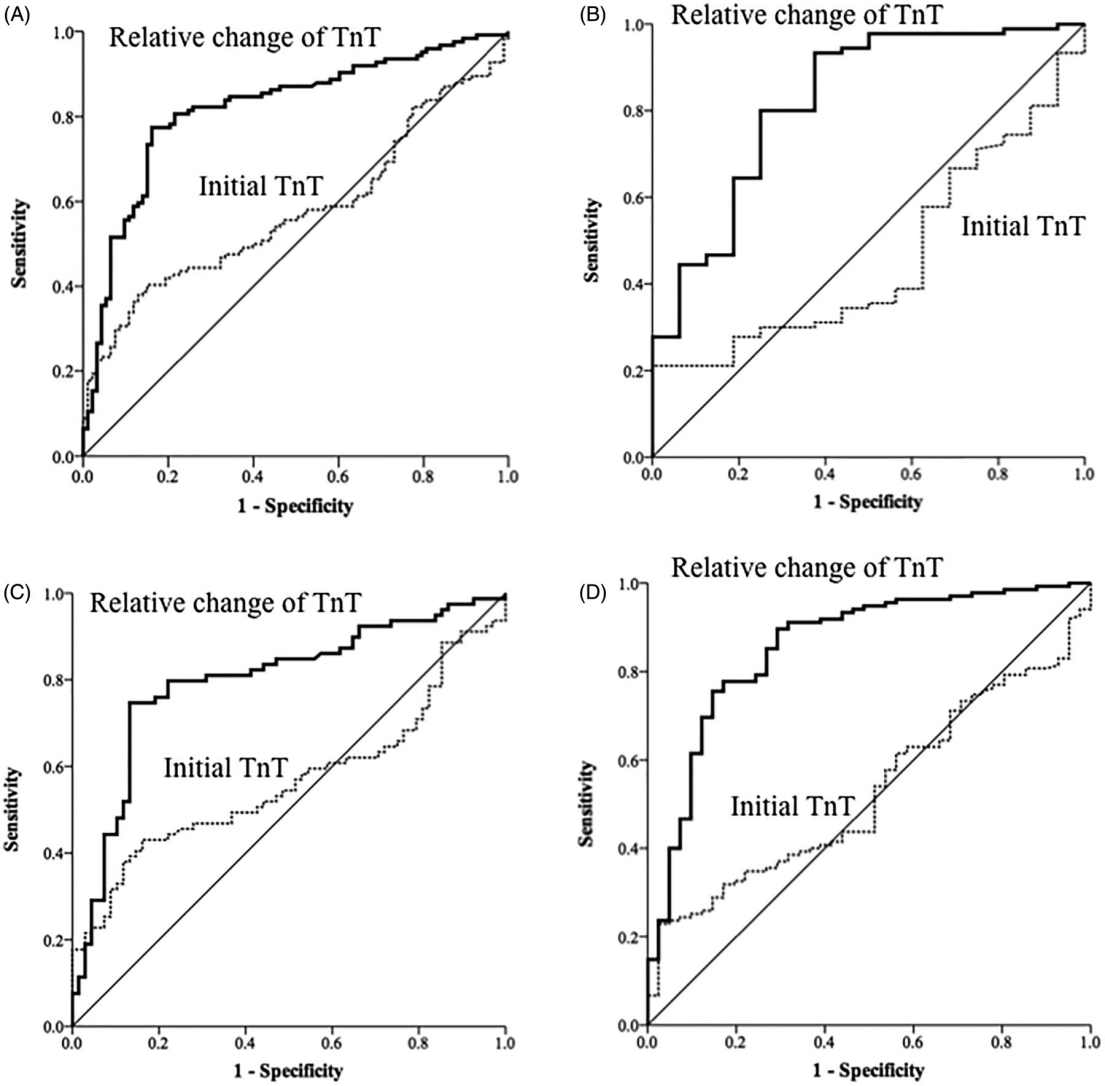
Receiver operating characteristic curves of high-sensitivity cardiac troponin-T (TnT) levels on admission, and dynamic changes in these levels for the diagnosis of acute myocardial infarction (diagnosis based on coronary angiography or cardiac echocardiography) (A) for patients with eGFR < 60 mL/min/1.73 m^2^, (B) patients with eGFR > 60 mL/min/1.73 m^2^, (C) patients with chronic kidney disease (CKD) history, and (D) patients without CKD history.

**Figure 3.**
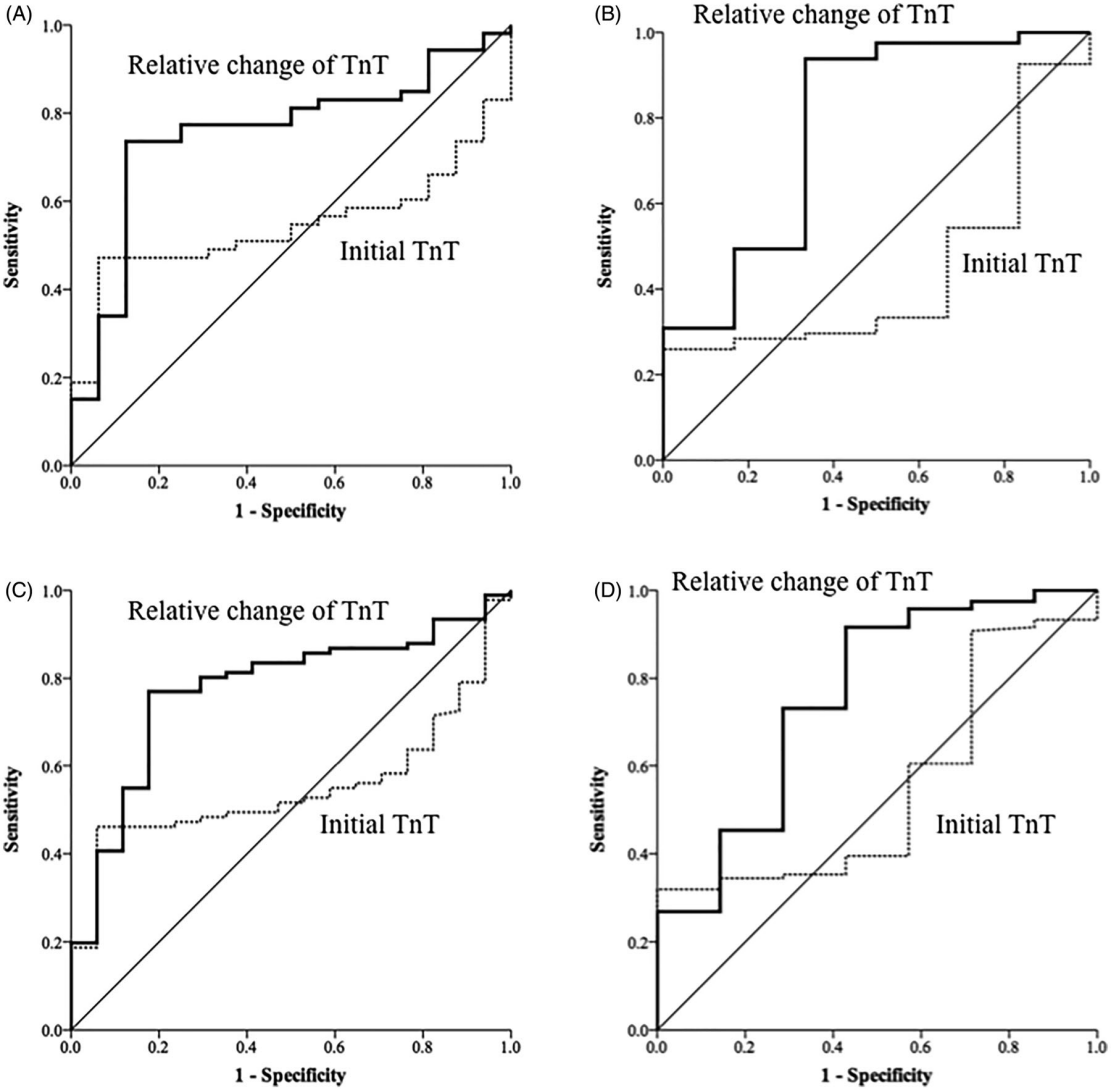
Receiver operating characteristic curves of high-sensitivity cardiac troponin-T (TnT) levels on admission and dynamic changes in these levels for the diagnosis of acute myocardial infarction (diagnosis based on coronary angiography only) (A) for patients with eGFR < 60 mL/min/1.73 m^2^, (B) patients with eGFR > 60 mL/min/1.73 m^2^, (C) patients with chronic kidney disease (CKD) history, and (D) patients without CKD history.

The AUC of the initial hs-cTnT levels for the diagnosis of AMI was 0.58 [95% confidence interval (CI), 0.5–0.65, *p* = 0.053] in patients with low eGFR and 0.54 (95% CI, 0.4–0.67, *p* = 0.612) in patients with normal eGFR (Figure 2(A,B)). The AUC of the relative changes of hs-cTnT for diagnosis of AMI was 0.82 (95% CI, 0.76–0.88, *p* < 0.001) in patients with low eGFR and 0.82 (95% CI, 0.71–0.91, *p* < 0.001) in patients with normal eGFR (Figure 2(A,B)). The AUC of initial hs-cTnT levels for diagnosis of AMI was 0.57 [95% confidence interval (CI), 0.47–0.66, *p* = 0.167] in CKD (+) group and 0.53 (95% CI, 0.44–0.62, *p* = 0.587) in CKD (−) group (Figure 2(C,D)). The AUC of the relative changes of hs-cTnT for the diagnosis of AMI was 0.80 (95% CI, 0.72–0.87, *p* < 0.001) in CKD (+) and 0.86 (95% CI, 0.79–0.93, *p* < 0.001) in CKD (−) (Figure 2(C,D)). The ROC curves of the initial hs-cTnT and the relative changes among 217 patients who underwent coronary angiography within 3 days of admission are shown in Figure 3. Making the diagnosis of AMI based on the relative changes of hs-cTnT was more accurate than using the just value of initial hs-cTnT (DeLong test, *p* < 0.001).

The diagnostic performances of various cutoff values for dynamic change in hs-cTnT are shown in Table 4. For the diagnosis of AMI based on coronary angiography or echocardiography and electrocardiography (*n* = 359), the optimal cutoff values of the relative changes in hs-cTnT were 16% for patients with low eGFR, 16% for CKD (+), 12% for patients with normal eGFR, and 11% for CKD (−) (Table 4). For the identification of AMI, a cutoff value of 16% for patients with low eGFR had a sensitivity of 77%, a specificity of 84%, a positive predictive value of 87%, and a negative predictive value of 73% for the identification of AMI (Table 4).

**Table 4.** Diagnostic performance of dynamic change for high-sensitivity cardiac troponin-T levels for the diagnosis of acute myocardial infarction.

Performance	eGF*R* < 60 mL/min/1.73 m^2^, *n* = 240 AUC (95% CI), 0.82 (0.76–0.88)			
Relative change in hs-cTnT	Sensitivity	Specificity	PPV	NPV
5%	0.85	0.66	0.79	0.77
9%	0.82	0.74	0.81	0.75
16%	0.77	0.84	0.87	0.73
25%	0.73	0.85	0.87	0.7

	eGF*R* ≥ 60 mL/min/1.73 m^2^, *n* = 119 AUC (95% CI), 0.82 (0.71–0.94)			
3%	0.96	0.5	0.9	0.73
7%	0.94	0.56	0.91	0.67
12%	0.93	0.62	0.92	0.59
48%	0.8	0.75	0.94	0.44

eGFR: estimated glomerular filtration rate; AUC: area under the receiver operating characteristic curve; hs-cTnT: high-sensitivity cardiac troponin-T; PPV: positive predictive value; NPV: negative predictive value.

For the diagnosis of AMI based on coronary angiography (*n* = 217), the optimal cutoff values of the relative changes in hs-cTnT were 16% for patients with low eGFR, 16% for CKD (+), 9% for patients with normal eGFR, and 8% for CKD (−) (Table 5).

**Table 5.** Optimal cutoff values for dynamic changes in high-sensitivity cardiac troponin-T levels for the diagnosis of acute myocardial infarction.

	Diagnosis of AMI based on coronary angiography or echocardiography			
Performance	eGF*R* < 60 mL/min/1.73 m^2^ (*n* = 240)	eGF*R* ≥ 60 mL/min/1.73 m^2^ (*n* = 119)	CKD history (*n* = 157)	No CKD history (*n* = 202)
Cutoff value	16%	12%	16%	11%
AUC (95% CI)	0.82 (0.76–0.88), *p* < 0.001	0.82 (0.71–0.94), *p* < 0.001	0.8 (0.72–0.87), *p* < 0.001	0.86 (0.79–0.93), *p* < 0.001
Sensitivity	0.77	0.93	0.83	0.9
Specificity	0.84	0.62	0.8	0.71

	Diagnosis of AMI based on coronary angiography only			
	eGF*R* < 60 mL/min/1.73 m^2^ (*n* = 119)	eGF*R* ≥ 60 mL/min/1.73 m^2^ (*n* = 98)	CKD history (*n* = 73)	No CKD history (*n* = 144)
Cutoff value	16%	9%	16%	8%
AUC (95% CI)	0.78 (0.66–0.89), *p* < 0.001	0.78 (0.56–1), *p* = 0.022	0.76 (0.62–0.89), *p* = 0.002	0.76 (0.56–0.96), *p* = 0.022
Sensitivity	0.77	0.94	0.74	0.92
Specificity	0.82	0.67	0.88	0.57

eGFR: estimated glomerular filtration rate; CKD: chronic kidney disease; AUC: area under the receiver operating characteristic curve.

In subgroup analyses, we divided patients into 5 groups according to eGFR (stage 1, eGFR ≥90 mL/min/1.73 m^2^; stage 2, eGFR 60–89 mL/min/1.73 m^2^; stage 3, eGFR 30–59 mL/min/1.73 m^2^; stage 4, eGFR 15–29 mL/min/1.73 m^2^; stage 5 eGFR <15 mL/min/1.73 m^2^ or under renal replacement therapy.), and divided patients into young and old groups by age of 70, with and without underlying CAD, and with and without underlying heart failure (Table 6). Overall, the relative changes in hs-cTnT had good diagnostic performance for AMI except for old patients with normal eGFR (AUC: 0.7, *p* value =0.076) and patients with underlying CAD and normal eGFR (AUC: 0.76, *p* value =0.205) (Table 6). The AUC of absolute changes of hs-cTnT for diagnosis of AMI was 0.83 (95% CI, 0.78–0.89, *p* < 0.001) in patients with low eGFR and 0.82 (95% CI, 0.72–0.93, *p* < 0.001) in patients with normal eGFR. Comparing the diagnostic performance of AMI by DeLong test, using the absolute and relative changes of hs-cTnT reached similar effects. Of the 186 patients with AMI who underwent coronary angiography, single-vessel involvement was observed in 49 patients (26.3%), two-vessel disease was seen in 51 (27.4%), triple-vessel disease was observed in 86 patients (46.2%), and left main coronary artery disease was found in 23 patients (12.4%).

**Table 6.** Subgroup analyses of initial and relative change of high-sensitivity cardiac troponin-T for diagnosis performance of acute myocardial infarction.

		Initial hs-cTnT		Relative change in hs-cTnT (%)				
	*n*	AUC (95% CI)	*p* Value	AUC (95% CI)	*p* Value	Optimal cutoff values	Sensitivity	Specificity
eGFR stages								
Stage 1	35	0.36 (0.11–0.6)	0.246	0.96 (0.88–1)	<0.001	9.5%	1	0.75
Stage 2	84	0.51 (0.35–0.67)	0.952	0.78 (0.63–0.93)	<0.001	12%	0.9	0.59
Stage 3	103	0.59 (0.47–0.7)	0.139	0.86 (0.78–0.94)	<0.001	19.6%	0.81	0.81
Stage 4	44	0.65 (0.47–0.82)	0.102	0.83 (0.69–0.97)	<0.001	15.6%	0.85	0.8
Stage 5	93	0.57 (0.45–0.69)	0.276	0.78 (0.68–0.88)	<0.001	17.8%	0.69	0.88
Ag*e* ≥ 70 y/o								
eGF*R* < 60 mL/min/1.73 m^2^	150	0.65 (0.56–0.74)	0.048	0.81 (0.74–0.89)	<0.001	11%	0.83	0.78
eGF*R* ≥ 60 ml/min/1.73 m^2^	40	0.65 (0.46–0.84)	0.186	0.7 (0.47–0.92)	0.076	N/A	N/A	N/A
Ag*e* < 70 y/o								
eGF*R* < 60 mL/min/1.73 m^2^	90	0.46 (0.33–0.58)	0.507	0.83 (0.74–0.92)	<0.001	17.8%	0.77	0.88
eGF*R* ≥ 60 ml/min/1.73 m^2^	79	0.36 (0.19–0.54)	0.271	0.91 (0.82–1)	0.001	9.4%	0.97	0.67
CAD history								
eGF*R* < 60 mL/min/1.73 m^2^	83	0.45 (0.32–0.59)	0.507	0.8 (0.69–0.91)	<0.001	17.8%	0.81	0.84
eGF*R* ≥ 60 ml/min/1.73 m^2^	26	0.78 (0.54–1)	0.089	0.76 (0.36–1)	0.205	N/A	N/A	N/A
No CAD history								
eGF*R* < 60 mL/min/1.73 m^2^	157	0.64 (0.55–0.73)	0.005	0.83 (0.76–0.9)	<0.001	16%	0.75	0.83
eGF*R* ≥ 60 ml/min/1.73 m^2^	93	0.37 (0.23–0.51)	0.072	0.85 (0.74–0.96)	<0.001	9%	0.97	0.58
Heart failure history								
eGF*R* < 60 mL/min/1.73 m^2^	82	0.61 (0.48–0.74)	0.113	0.87 (0.79–0.96)	<0.001	11.2%	0.92	0.82
eGF*R* ≥ 60 ml/min/1.73 m^2^	15	N/A*	N/A*	N/A*	N/A*	N/A*	N/A*	N/A*
No heart failure history								
eGF*R* < 60 mL/min/1.73 m^2^	158	0.56 (0.47–0.66)	0.218	0.8 (0.73–0.88)	<0.001	16%	0.72	0.83
eGF*R* ≥ 60 ml/min/1.73 m^2^	104	0.46 (0.32–0.6)	0.597	0.83 (0.72–0.95)	<0.001	12%	0.94	0.63

Definition of eGFR stages: stage 1, eGFR ≥90 mL/min; stage 2, eGFR 60–89 mL/min; stage 3, eGFR 30–59 mL/min; stage 4, eGFR 15–29 mL/min; stage 5 eGFR <15 mL/min or under renal replacement therapy. Optimal cutoff value was defined as highest value of combination of sensitivity and specificity.*All patients diagnosed with acute myocardial infarction.

AUC: area under the receiver operating characteristic curve; CAD: coronary artery disease; eGFR: estimated glomerular filtration rate; hs-cTnT: high-sensitivity cardiac troponin-T; PPV: positive predictive value; NPV: negative predictive value; N/A: Not applicable; y/o: years old.

## Discussion

In this prospective observational study, we demonstrated that the diagnostic accuracy of initial hs-cTnT levels for the diagnosis of AMI is low regardless of renal function [AUC, 0.58 for low eGFR; AUC, 0.54 for normal eGFR; AUC, 0.57 for CKD (+) and AUC, 0.53 for CKD (−)] (Figure 2). Subsequent measurements of hs-cTnT 3 h later improved the diagnostic accuracy, and the optimal cutoff values differed according to different levels of kidney function. The study revealed that for the diagnosis of AMI in patients with low eGFR, relative changes in hs-cTnT levels of 16% yielded a sensitivity of 77%, a specificity of 84%, a positive predictive value of 87%, and a negative predictive value of 73% (Table 3). For patients with normal eGFR, the optimal cutoff value was 12% with a sensitivity of 93%, a specificity of 62%, a positive predictive value of 92%, and a negative predictive value of 59% (Table 3). When stratifying patients by with and without preexisting CKD, the results for optimal cutoff values of relative changes in hs-cTnT levels were similar [16% for CKD (+); 11% for CKD (−)]. In the subgroup analysis of patients who underwent coronary angiography (*n* = 217), the optimal cutoff values for patients with low eGFR and CKD (+) were both 16%. The findings of the study highlight the importance of relative changes in hs-cTnT in patients with impaired renal function for the diagnosis of AMI.

Elevation of hs-cTnT is often detected in patients with renal failure, heart failure, myocarditis, ventricular hypertrophy, arrhythmias, pulmonary embolism, and after coronary procedures and seizures [1,16–18]. Troponin-T is also released from cells other than myocardial cells. One clinical study shows that troponin-T is expressed in the skeletal muscle of dialysis patients [19], and this expression could explain the mechanism of elevated troponin-T in patients with seizure or rhabdomyolysis [17,20]. Although elevation of cardiac troponins in the blood usually reflects injury leading to necrosis of the myocardium, such levels do not indicate the mechanism of ischemia-induced myocardial necrosis [1]. According to the Fourth Universal Definition of Myocardial Infarction Expert Consensus Document of myocardial infarction, measurements of cardiac troponins should be repeated 3–6 h thereafter because chronic elevation of cardiac troponin is noted in patients with renal failure [1].

The study revealed that fewer of the patients with lower eGFR presented initially with typical chest pain than patients with normal eGFR (43.3 versus 73.9%). A previous report that included 29,319 patients with advanced CKD with AMI also showed different presentations of chest pain depending on the presence of CKD (40.4% in patients with CKD versus 61.6% for patients without CKD) [7]. As a consequence, the diagnosis of AMI in patients with CKD strongly depends on cardiac troponin levels because these patients usually present with atypical AMI symptoms. However, elevated cardiac troponin can be detected in 33–43% of patients with CKD [21]. The detection of elevated troponin T reflects a variety of cardiovascular pathophysiologies other than just impaired renal clearance, including uremic cardiomyopathy, ventricular dysfunction, left ventricular hypertrophy, anemia, inflammation, and endothelial dysfunction [21]. As a result, chronically elevated troponin can act as a prognostic factor in patients with CKD [21,22]. One study showed that the normal range of serum hs-cTnT in patients with CKD is higher than that in patients without CKD (139 ng/L in patients with CKD versus 14 ng/L in patients without CKD) [23]. Another study revealed that the baseline hs-cTnT levels in asymptomatic hemodialysis patients were higher than non-CKD patients (54.3 versus 18 ng/L) [24]. Nevertheless, the Fourth Universal Definition of myocardial infarction used the same value of hs-cTnT for the diagnosis of AMI in patients either with reduced or normal eGFR.

Previous studies demonstrates that hs-cTnT increases gradually with time after onset of symptoms and the diagnostic performance was excellent in patients within 2 h after the onset of symptoms [25–27]. The median time between sampling of hs-cTnT and onset of symptoms was 3 h in our study. However, the diagnostic accuracy of the initial hs-cTnT levels for the diagnosis of AMI in patients with renal insufficiency is poor, with an AUC of 0.58, which was consistent with the result of a study in 2013 that showed an AUC of 0.61 [28]. Some studies have indicated that the use of the dynamic change in hs-cTnT increases the diagnostic accuracy for AMI in patients with CKD [29–32]. A study with 670 patients (undergoing regular hemodialysis presented with chest pain or dyspnea) proposed the optimal cutoff value of the relative changes in hs-cTnT after 3 h was 24% for the diagnosis of AMI [29]. Recently, a large prospective study showed that a relative change in hs-cTnT of 250% after 3 h could be used to rule in AMI, with a positive predictive value of 0.8 in patients with CKD who presented with chest pain [31]. Most studies enrolled patients with CKD or end-stage renal disease (ESRD) with typical chest pain. However, more than one-half of AMI patients with CKD present with atypical symptoms other than chest pain [7]. Thus, we enrolled patients with typical chest pain and other atypical symptoms such as dyspnea, upper abdominal pain, and cardiac arrest.

Ascertainment of the final diagnosis of AMI is a critical issue. Previous studies have adjudicated the final diagnosis based on clinical, laboratory, and imaging findings by cardiologists [2,29,31]. However, we defined AMI clearly and objectively according to image findings (coronary angiography or echocardiography combined with electrocardiography). Reiter M et al. found that hs-cTnT has high diagnostic accuracy also in the elderly and the optimal cutoff levels are higher in older as compared with younger patients [33]. However, in patients with low eGFR, our study showed that the optimal cutoff level of relative changes of hs-cTnT is lower in the old group compared with the young group (11 versus 17.8). Small number of cases (*N* = 40) might result in poor diagnostic performance of relative changes of hs-cTnT in the old group with normal eGFR (AUC: 0.7, *p* value =0.076) (Table 6). Reiter M *et al.* also showed that the optimal cutoff levels tend to be higher in patients with preexisting CAD using hs-cTnT [34]. In consistence with previous study, we found that the optimal cutoff level of relative changes of hs-cTnT were higher in patients with low eGFR and preexisting CAD than those with low eGFR and without preexisting CAD (17.8 versus 16%) (Table 6). Small number of cases (*N* = 26) might result in statistical insignificance of diagnostic performance of relative changes of hs-cTnT among patients with normal eGFR and preexisting CAD (AUC: 0.76, *p* value =0.205) (Table 6).

There are several limitations to our study. First, initial serum creatinine was used to calculate eGFR. However, the calculated eGFR does not represent chronic status. It could be a result of coexisting acute kidney injury (AKI), which is common in AMI patients induced by heart failure. In our stratified analysis, two groups (with and without CKD) yielded similar results. Hence, using initial creatinine for eGFR calculation didn’t seem to interfere our study results, and it is easy and suitable for clinical application. Second, we also enrolled 93 patients with ST-segment elevation myocardial infarction (STEMI), in which 81 of them underwent coronary angiography. The relative changes in hs-cTnT may be confounded by percutaneous coronary intervention–induced myocardial injury. Third, the enrollment of our patients was confined in the CCU; the patients with similar clinical presentation but spared being sent into CCU were not recruited. In the future, if simultaneous coronary angiography (or/and echocardiography) and serial hs-cTnT measurements can be more universally performed in suspected AMI patients, further progress may be made in the research of the clinical application of hs-cTnT.

In conclusion, using initial hs-cTnT for diagnosis of AMI in patients with renal insufficiency had poor diagnostic accuracy. Using relative changes in hs-cTnT 3 h after the initial level yielded better results for the detection of AMI. The optimal cutoff values for patients with low eGFR was 16%, with a sensitivity of 77% and specificity of 84%; while the optimal cutoff values was 12% for patients with normal eGFR, with a sensitivity of 93% and a specificity of 62%. The results of the study could offer new horizons to assess cardiac enzymes in renal-insufficient patients and could offer a new and simple method to evaluate renal-insufficient patients with possible AMI, prompting timely diagnosis and treatment for AMI, meanwhile, reducing the number of falsely diagnosed AMI if merely using initial hs-cTnT.
